# Supplementary material for: Widespread selection and gene flow shape the genomic landscape during a radiation of monkeyflowers
Source: PLoS Biol. 2019 Jul 24;17(7):e3000391. doi: 10.1371/journal.pbio.3000391 (PMC6660095; doi:10.1371/journal.pbio.3000391)
Supplement: S1 Table — The table includes map length in cM for each LG, the number of markers associated with each LG, the number of unique map positions, and the average genetic distance in cM between each unique map position. SDs are given in parentheses. LG, linkage group. (DOCX) [file pbio.3000391.s001.docx]

| LG | Map length (cM) | Number of markers | Unique map positions | Avg. genetic dist. between unique markers in cM (sd) |
| --- | --- | --- | --- | --- |
| 1 | 93.9 | 969 | 335 | 0.28 (0.33) |
| 2 | 71.37 | 893 | 253 | 0.28 (0.88) |
| 3 | 76.3 | 912 | 256 | 0.30 (0.65) |
| 4 | 70.2 | 851 | 257 | 0.28 (0.41) |
| 5 | 78.7 | 778 | 295 | 0.27 (0.29) |
| 6 | 69.1 | 741 | 247 | 0.28 (0.88) |
| 7 | 59.8 | 738 | 234 | 0.26 (0.33) |
| 8 | 65.6 | 674 | 246 | 0.27 (0.41) |
| 9 | 68.6 | 623 | 182 | 0.37 (0.58) |
| 10 | 71.1 | 410 | 150 | 0.48 (0.96) |
| Avg. | 72.74 (8.69) | 758.90 (155.75) | 245.50 (49.04) | 0.31 (0.06) |
