# Supplementary material for: Widespread selection and gene flow shape the genomic landscape during a radiation of monkeyflowers
Source: PLoS Biol. 2019 Jul 24;17(7):e3000391. doi: 10.1371/journal.pbio.3000391 (PMC6660095; doi:10.1371/journal.pbio.3000391)
Supplement: S2 Table — The number and percent of core genes found in the final assembly are shown for each analysis (CEGMA, n = 248; BUSCO, n = 1,440). (DOCX) [file pbio.3000391.s002.docx]

| Analysis | # Genes | % Found in Assembly |
| --- | --- | --- |
| CEGMA Complete | 233 | 93.95 |
| CEGMA Partial | 244 | 98.39 |
| BUSCO total complete (duplicated) | 1340 (61) | 93 (4.2) |
| BUSCO Fragmented | 29 | 2.0 |
| BUSCO Missing | 71 | 5.0 |
