# Supplementary material for: Widespread selection and gene flow shape the genomic landscape during a radiation of monkeyflowers
Source: PLoS Biol. 2019 Jul 24;17(7):e3000391. doi: 10.1371/journal.pbio.3000391 (PMC6660095; doi:10.1371/journal.pbio.3000391)
Supplement: S3 Table — Includes their taxon identity, sampling location, percent read alignment, and average sequencing depth. (DOCX) [file pbio.3000391.s003.docx]

| Sample | Taxon | Latitude | Longitude | % Reads aligned | Seq.  Depth |
| --- | --- | --- | --- | --- | --- |
| 159_83 | ssp. *aridus* | 32.6630 | -116.2230 | 91.7 | 21.12 |
| 159_84 | ssp. *aridus* | 32.6630 | -116.2230 | 89.3 | 21.98 |
| 195_1 | ssp. *aridus* | 32.6300 | -116.1429 | 92.6 | 20.20 |
| T84 | ssp. *aridus* | 32.6526 | -116.2449 | 87.2 | 21.75 |
| T102 | ssp. *aurantiacus* | 39.0424 | -122.7727 | 94.9 | 23.74 |
| T104 | ssp. *aurantiacus* | 39.2045 | -123.7646 | 94.6 | 25.09 |
| T50 | ssp. *aurantiacus* | 35.9865 | -121.4928 | 88.3 | 24.36 |
| T92 | ssp. *aurantiacus* | 37.8459 | -120.6110 | 94.0 | 15.16 |
| T144 | ssp. *calycinus* | 34.1929 | -117.2784 | 93.2 | 26.00 |
| T150 | ssp. *calycinus* | 33.8564 | -116.8481 | 94.7 | 24.02 |
| T90 | ssp. *calycinus* | 35.5918 | -118.5052 | 91.3 | 19.97 |
| T91 | ssp. *calycinus* | 35.3172 | -118.5871 | 95.5 | 27.91 |
| T101 | ssp. *grandiflorus* | 39.5536 | -121.4301 | 92.0 | 16.05 |
| T61 | ssp. *grandiflorus* | 39.5590 | -120.8243 | 91.6 | 17.31 |
| T96 | ssp. *grandiflorus* | 39.0122 | -120.7552 | 92.0 | 28.21 |
| T99 | ssp. *grandiflorus* | 39.4376 | -121.0599 | 91.4 | 23.84 |
| DPR | ssp. *longiflorus* | 33.7459 | -117.4485 | 96.0 | 26.88 |
| SS | ssp. *longiflorus* | 34.2722 | -118.6100 | 94.2 | 30.86 |
| T33 | ssp. *longiflorus* | 34.3438 | -118.5099 | 94.6 | 18.87 |
| T8 | ssp. *longiflorus* | 34.1347 | -118.6452 | 82.6 | 25.11 |
| KK168 | ssp. *parviflorus* | 34.0180 | -119.6730 | 91.8 | 23.66 |
| KK161 | ssp. *parviflorus* | 34.0180 | -119.6730 | 92.0 | 19.11 |
| KK180 | ssp. *parviflorus* | 34.0180 | -119.6730 | 92.4 | 18.18 |
| KK182 | ssp. *parviflorus* | 34.0193 | -119.6802 | 91.3 | 19.46 |
| ELF | ssp. *puniceus*, red | 33.0860 | -117.1453 | 93.0 | 18.20 |
| JMC | ssp. *puniceus*, red | 32.7373 | -116.9541 | 93.8 | 19.06 |
| LH | ssp. *puniceus*, red | 33.0609 | -117.1188 | 87.1 | 19.77 |
| MT | ssp. *puniceus*, red | 32.8210 | -117.0618 | 93.7 | 20.85 |
| UCSD | ssp. *puniceus*, red | 32.8894 | -117.2362 | 87.0 | 18.23 |
| BCRD | ssp. *puniceus*, yellow | 32.9496 | -116.6380 | 94.6 | 20.85 |
| INJ | ssp. *puniceus*, yellow | 33.0979 | -116.6643 | 93.1 | 18.83 |
| LO | ssp. *puniceus*, yellow | 32.6767 | -116.3312 | 93.4 | 18.04 |
| PCT | ssp. *puniceus*, yellow | 32.7326 | -116.4698 | 92.3 | 19.68 |
| POTR | ssp. *puniceus*, yellow | 32.6038 | -116.6339 | 90.5 | 19.27 |
| CLV_GH | *M. clevelandii* | 33.1589 | -116.8122 | 92.3 | 21.31 |
| CLV_11 | *M. clevelandii* | 33.3391 | -116.9325 | 84.4 | 15.52 |
| CLV_4 | *M. clevelandii* | 33.3391 | -116.9325 | 89.3 | 17.31 |
