# Supplementary material for: Widespread selection and gene flow shape the genomic landscape during a radiation of monkeyflowers
Source: PLoS Biol. 2019 Jul 24;17(7):e3000391. doi: 10.1371/journal.pbio.3000391 (PMC6660095; doi:10.1371/journal.pbio.3000391)
Supplement: S4 Table — PC1, first principal component. (DOCX) [file pbio.3000391.s004.docx]

| Comparison | *F_ST_* | *d_xy_* PC1 |
| --- | --- | --- |
| Aur_Ari | 0.85707 | 0.95575 |
| Gra_Ari | 0.89606 | 0.86182 |
| Cal_Ari | 0.88738 | 0.95423 |
| CLV_Ari | 0.8969 | 0.39278 |
| Par_Ari | 0.85342 | 0.94563 |
| Lon_Ari | 0.9046 | 0.96057 |
| Aur_Gra | 0.81191 | 0.85563 |
| Aur_Cal | 0.32407 | 0.90376 |
| Aur_CLV | 0.90725 | 0.46698 |
| Aur_Par | 0.79434 | 0.94778 |
| Lon_Aur | 0.40962 | 0.90381 |
| Ari_Y | 0.90097 | 0.94262 |
| Aur_Y | 0.451 | 0.90512 |
| Gra_Y | 0.91203 | 0.90305 |
| Cal_Y | 0.49255 | 0.88634 |
| CLV_Y | 0.90576 | 0.54264 |
| Par_Y | 0.86511 | 0.94318 |
| Lon_Y | 0.59046 | 0.88521 |
| Cal_Gra | 0.89043 | 0.88319 |
| CLV_Gra | 0.91181 | 0.32045 |
| Par_Gra | 0.89579 | 0.86769 |
| Lon_Gra | 0.88549 | 0.89344 |
| Cal_Par | 0.83543 | 0.94124 |
| Lon_Cal | 0.3786 | 0.87337 |
| Cal_CLV | 0.90548 | 0.52607 |
| Par_CLV | 0.91991 | 0.45233 |
| Lon_CLV | 0.90738 | 0.51482 |
| Lon_Par | 0.84197 | 0.94507 |
| R_Ari | 0.90783 | 0.94574 |
| R_Aur | 0.51788 | 0.89861 |
| R_Y | 0.51423 | 0.84798 |
| R_Gra | 0.91936 | 0.90695 |
| R_Cal | 0.62051 | 0.88316 |
| R_CLV | 0.90758 | 0.55149 |
| R_Par | 0.87615 | 0.93825 |
| R_Lon | 0.66912 | 0.88264 |

| Taxon | π PC1 |
| --- | --- |
| Ari | 0.9251 |
| Aur | 0.86508 |
| Cal | 0.96734 |
| CLV | 0.92017 |
| Gra | 0.77181 |
| Lon | 0.95166 |
| Par | 0.91044 |
| R | 0.97282 |
| Y | 0.97255 |
