# Supplementary material for: Widespread selection and gene flow shape the genomic landscape during a radiation of monkeyflowers
Source: PLoS Biol. 2019 Jul 24;17(7):e3000391. doi: 10.1371/journal.pbio.3000391 (PMC6660095; doi:10.1371/journal.pbio.3000391)
Supplement: S5 Table — (DOCX) [file pbio.3000391.s005.docx]

| Variables | Pearson’s r | Regression equation | *p* |
| --- | --- | --- | --- |
| *d_xy_* & π | -0.93 | y = -60.5*x* + 1.33 | < 0.001 |
| π & gene count | 0.59 | y = 3.9*x* - 0.85 | 0.130 |
| π & cM/mbp | -0.79 | y = -5.4*x* + 0.46 | 0.020 |
| *F_ST_* & π | -0.89 | y = -53.3*x* - 0.13 | 0.003 |
| *F_ST_* & gene count | 0.81 | y = 46.5*x* + 0.04 | 0.016 |
| *F_ST_* & cM/mbp | -0.73 | y = -25.5*x* + 0.01 | 0.041 |
