# Supplementary material for: Widespread selection and gene flow shape the genomic landscape during a radiation of monkeyflowers
Source: PLoS Biol. 2019 Jul 24;17(7):e3000391. doi: 10.1371/journal.pbio.3000391 (PMC6660095; doi:10.1371/journal.pbio.3000391)
Supplement: S6 Table — (N = 10,000). (DOCX) [file pbio.3000391.s006.docx]

| Class | Mean Ns of deleterious mutations | Percent of deleterious mutations | Mean Ns of beneficial mutations | Percent of beneficial mutations | Migration rate (Nm) |
| --- | --- | --- | --- | --- | --- |
| Neutral | - | - | - | - | - |
| BGS | -10 | 5% | - | - | - |
|  | -10 | 10% | - | - | - |
|  | -100 | 5% | - | - | - |
|  | -100 | 10% | - | - | - |
| BDMI | -10 | 5% | - | - | 0.1 |
|  | -10 | 10% | - | - | 1 |
|  | -10 | 5% | - | - | 0.1 |
|  | -10 | 10% | - | - | 1 |
|  | -100 | 5% | - | - | 0.1 |
|  | -100 | 10% | - | - | 1 |
|  | -100 | 5% | - | - | 0.1 |
|  | -100 | 10% | - | - | 1 |
| Positive | - | - | 100 | 0.1% | - |
|  | - | - | 100 | 0.5% | - |
| Local adaptation | - | - | 100 | 0.1% | 0.1 |
|  | - | - | 100 | 0.1% | 1 |
|  | - | - | 100 | 0.5% | 0.1 |
|  | - | - | 100 | 0.5% | 1 |
| BGS & positive | -10 | 5% | 100 | 0.1% | - |
|  | -10 | 5% | 100 | 0.5% | - |
|  | -10 | 10% | 100 | 0.1% | - |
|  | -10 | 10% | 100 | 0.5% | - |
|  | -100 | 5% | 100 | 0.1% | - |
|  | -100 | 5% | 100 | 0.5% | - |
|  | -100 | 10% | 100 | 0.1% | - |
|  | -100 | 10% | 100 | 0.5% | - |
