# Supplementary material for: Widespread selection and gene flow shape the genomic landscape during a radiation of monkeyflowers
Source: PLoS Biol. 2019 Jul 24;17(7):e3000391. doi: 10.1371/journal.pbio.3000391 (PMC6660095; doi:10.1371/journal.pbio.3000391)
Supplement: S7 Table — Measures of Patterson’s D and the admixture proportion, f, are given for all 48 possible pairs of 4-taxon tests, with M. clevelandii as the out-group for each test. All values of Patterson’s D are statistically significant (P < 0.0001) based on a block jackknife approach. (DOCX) [file pbio.3000391.s007.docx]

| P1 | P2 | P3 | Patterson’s D | Admixture proportion |
| --- | --- | --- | --- | --- |
| Aur | Cal | Ari | 0.0808 | 0.0209 |
| Lon | Cal | Ari | 0.0233 | 0.0055 |
| Aur | Lon | Ari | 0.0633 | 0.0155 |
| Aur | R | Ari | 0.1360 | 0.0389 |
| Cal | R | Ari | 0.0651 | 0.0184 |
| Lon | R | Ari | 0.0883 | 0.0238 |
| Aur | Y | Ari | 0.1595 | 0.0474 |
| Cal | Y | Ari | 0.0949 | 0.0270 |
| Lon | Y | Ari | 0.1177 | 0.0324 |
| R | Y | Ari | 0.0371 | 0.0088 |
| R | Cal | Aur | 0.0831 | 0.0903 |
| Y | Cal | Aur | 0.0976 | 0.1034 |
| R | Lon | Aur | 0.0866 | 0.0909 |
| Y | Lon | Aur | 0.1009 | 0.1041 |
| Y | R | Aur | 0.0188 | 0.0145 |
| Ari | Par | Gra | 0.1053 | 0.0243 |
| Ari | Aur | Gra | 0.1821 | 0.0477 |
| Cal | Aur | Gra | 0.0648 | 0.0096 |
| Lon | Aur | Gra | 0.0936 | 0.0141 |
| Y | Aur | Gra | 0.1392 | 0.0218 |
| R | Aur | Gra | 0.1471 | 0.0222 |
| Par | Aur | Gra | 0.1072 | 0.0240 |
| Ari | Cal | Gra | 0.1523 | 0.0384 |
| Lon | Cal | Gra | 0.0344 | 0.0045 |
| Y | Cal | Gra | 0.0886 | 0.0123 |
| R | Cal | Gra | 0.0943 | 0.0126 |
| Par | Cal | Gra | 0.0665 | 0.0145 |
| Ari | Lon | Gra | 0.1371 | 0.0341 |
| Par | Lon | Gra | 0.0478 | 0.0101 |
| Y | Lon | Gra | 0.0589 | 0.0078 |
| R | Lon | Gra | 0.0650 | 0.0082 |
| Ari | R | Gra | 0.1072 | 0.0261 |
| Par | R | Gra | 0.0099 | 0.0019 |
| Ari | Y | Gra | 0.1103 | 0.0265 |
| Par | Y | Gra | 0.0127 | 0.0023 |
| R | Y | Gra | 0.0059 | 0.0004 |
| R | Y | Cal | 0.0149 | 0.0438 |
| Aur | Cal | Par | 0.0415 | 0.0182 |
| R | Cal | Par | 0.0104 | 0.0055 |
| Y | Cal | Par | 0.0176 | 0.0077 |
| Aur | Lon | Par | 0.0456 | 0.0207 |
| Cal | Lon | Par | 0.0049 | 0.0025 |
| R | Lon | Par | 0.0151 | 0.0080 |
| Y | Lon | Par | 0.0222 | 0.0102 |
| Aur | R | Par | 0.0311 | 0.0128 |
| Y | R | Par | 0.0090 | 0.0022 |
| Aur | Y | Par | 0.0240 | 0.0106 |
| R | Y | Lon | 0.0001 | -0.0018 |
