# Supplementary material for: Widespread selection and gene flow shape the genomic landscape during a radiation of monkeyflowers
Source: PLoS Biol. 2019 Jul 24;17(7):e3000391. doi: 10.1371/journal.pbio.3000391 (PMC6660095; doi:10.1371/journal.pbio.3000391)
Supplement: S8 Table — Pearson’s r was calculated between PC1 FST and fd for each of the 48 four-taxon tests, measured in 500-kb nonoverlapping windows. M. clevelandii is the out-group for each test. PC1, first principal component. (DOCX) [file pbio.3000391.s008.docx]

| P1 | P2 | P3 | Pearson's r | *p* |
| --- | --- | --- | --- | --- |
| Aur | Y | Par | -0.0682 | 0.1809 |
| R | Lon | Aur | -0.1645 | 0.0012 |
| Y | Cal | Aur | -0.1388 | 0.0062 |
| Ari | Cal | Gra | -0.3383 | 8.19E-12 |
| Y | Aur | Gra | -0.3126 | 3.23E-10 |
| Cal | R | Ari | -0.4135 | 2.06E-17 |
| Cal | Lon | Par | -0.1024 | 0.0441 |
| R | Y | Gra | -0.2732 | 4.75E-08 |
| Cal | Aur | Gra | -0.2313 | 4.28E-06 |
| Y | Cal | Gra | -0.2318 | 4.08E-06 |
| Y | Lon | Par | -0.2484 | 7.51E-07 |
| Aur | Y | Ari | -0.5850 | 6.54E-37 |
| R | Lon | Gra | -0.2797 | 2.18E-08 |
| Y | Lon | Aur | -0.1870 | 0.0002 |
| Par | Aur | Gra | -0.3334 | 1.67E-11 |
| Ari | Aur | Gra | -0.3344 | 1.45E-11 |
| Y | R | Par | -0.2681 | 8.58E-08 |
| R | Y | Cal | -0.0093 | 0.8546 |
| R | Aur | Gra | -0.3389 | 7.40E-12 |
| Ari | Y | Gra | -0.2782 | 2.61E-08 |
| Ari | Lon | Gra | -0.3538 | 7.45E-13 |
| Aur | Lon | Ari | -0.3056 | 8.24E-10 |
| Aur | R | Par | -0.0897 | 0.0779 |
| Par | Y | Gra | -0.2413 | 1.56E-06 |
| Aur | Cal | Ari | -0.3456 | 2.68E-12 |
| R | Lon | Par | -0.2026 | 5.96E-05 |
| Lon | Cal | Ari | -0.2312 | 4.29E-06 |
| Y | Lon | Gra | -0.2408 | 1.65E-06 |
| Par | Cal | Gra | -0.3394 | 6.91E-12 |
| R | Y | Lon | 0.0104 | 0.8381 |
| Lon | Y | Ari | -0.4775 | 1.94E-23 |
| Ari | Par | Gra | -0.2272 | 6.35E-06 |
| Lon | R | Ari | -0.4144 | 1.71E-17 |
| Aur | R | Ari | -0.5208 | 2.77E-28 |
| Aur | Lon | Par | -0.1015 | 0.0460 |
| Ari | R | Gra | -0.2636 | 1.42E-07 |
| Lon | Aur | Gra | -0.1697 | 0.0008 |
| Cal | Y | Ari | -0.4707 | 9.75E-23 |
| Par | Lon | Gra | -0.3104 | 4.33E-10 |
| Lon | Cal | Gra | -0.1296 | 0.0107 |
| R | Y | Ari | -0.3998 | 2.75E-16 |
| Aur | Cal | Par | -0.1359 | 0.0074 |
| R | Cal | Aur | -0.0881 | 0.0836 |
| R | Cal | Gra | -0.2463 | 9.31E-07 |
| Y | R | Aur | -0.1112 | 0.0287 |
| R | Cal | Par | -0.1981 | 8.75E-05 |
| Par | R | Gra | -0.2533 | 4.45E-07 |
| Y | Cal | Par | -0.2735 | 4.58E-08 |
