# Supplementary figures and images for: Widespread selection and gene flow shape the genomic landscape during a radiation of monkeyflowers
Source: PLoS Biol. 2019 Jul 24;17(7):e3000391. doi: 10.1371/journal.pbio.3000391 (PMC6660095; doi:10.1371/journal.pbio.3000391)

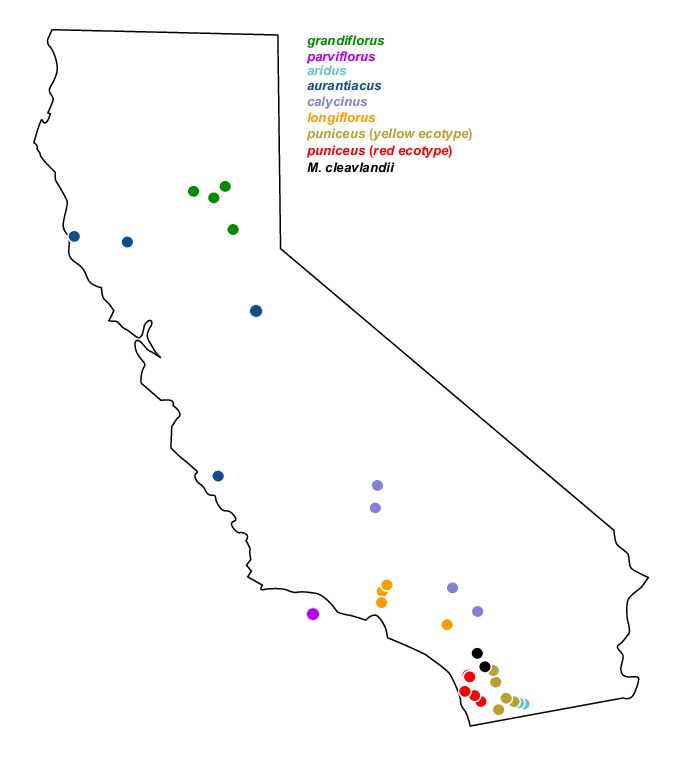

Supplement: S2 Fig — Detailed position information for each population can be found in S3 Table. (JPG) [file pbio.3000391.s010.jpg]

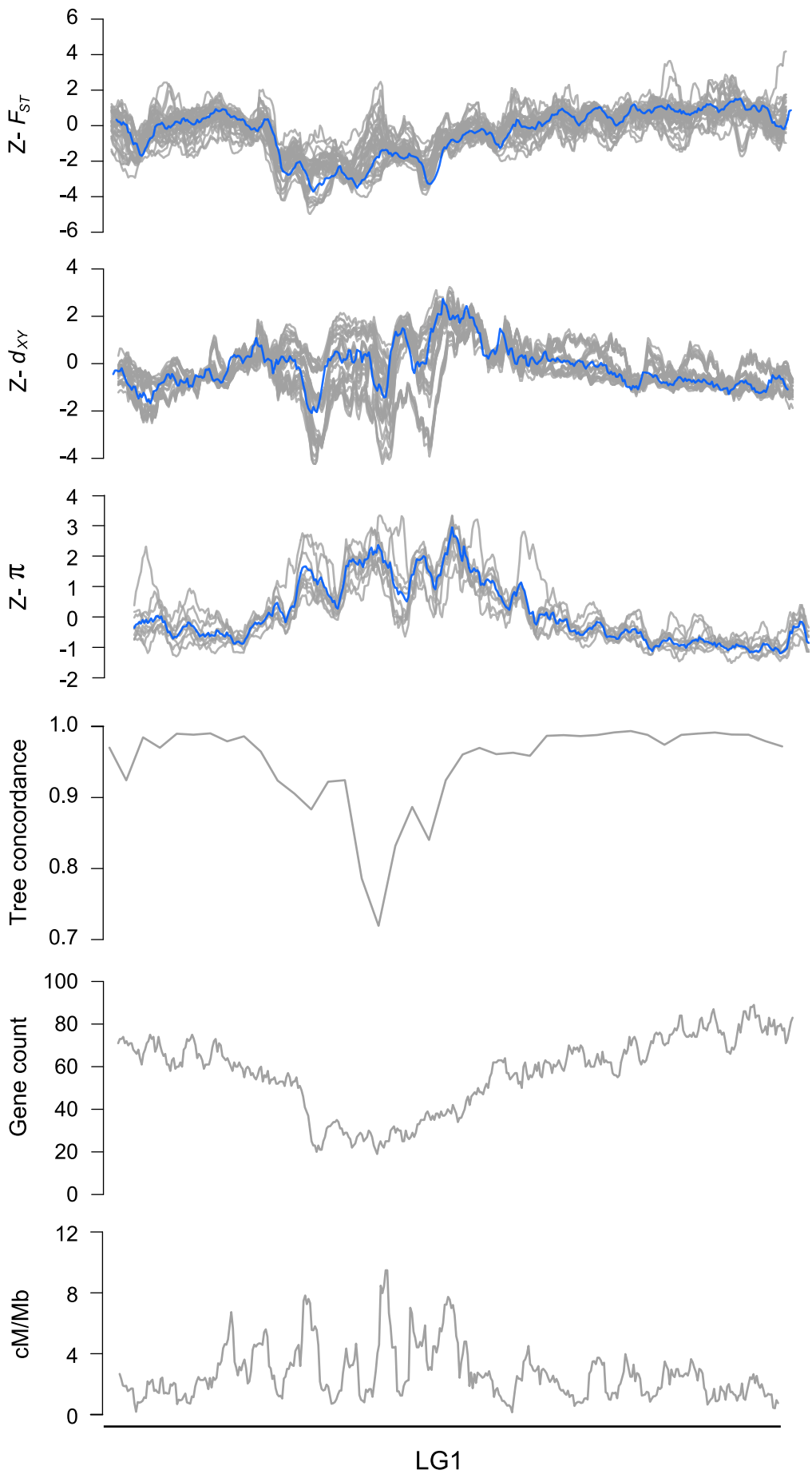

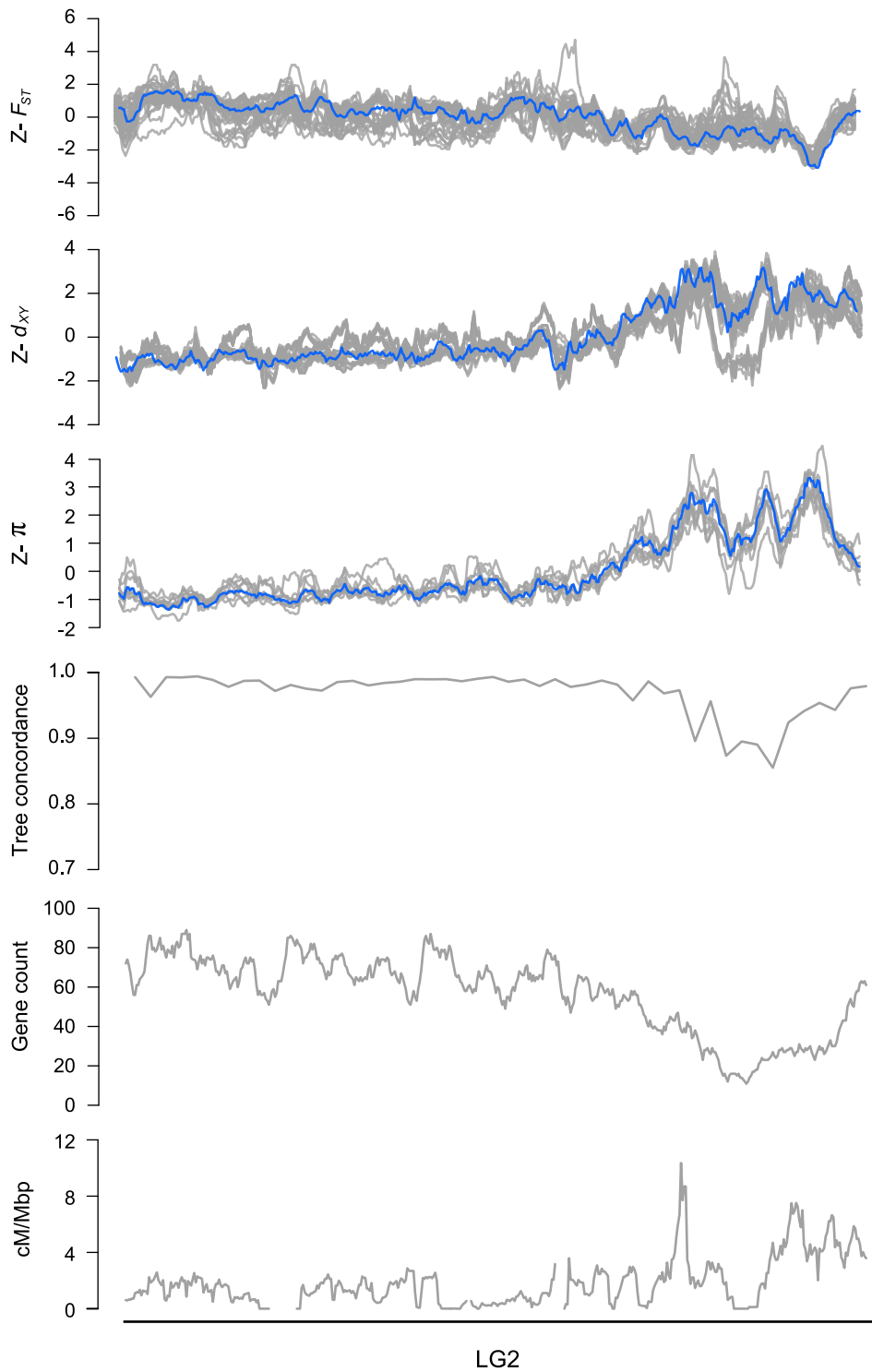

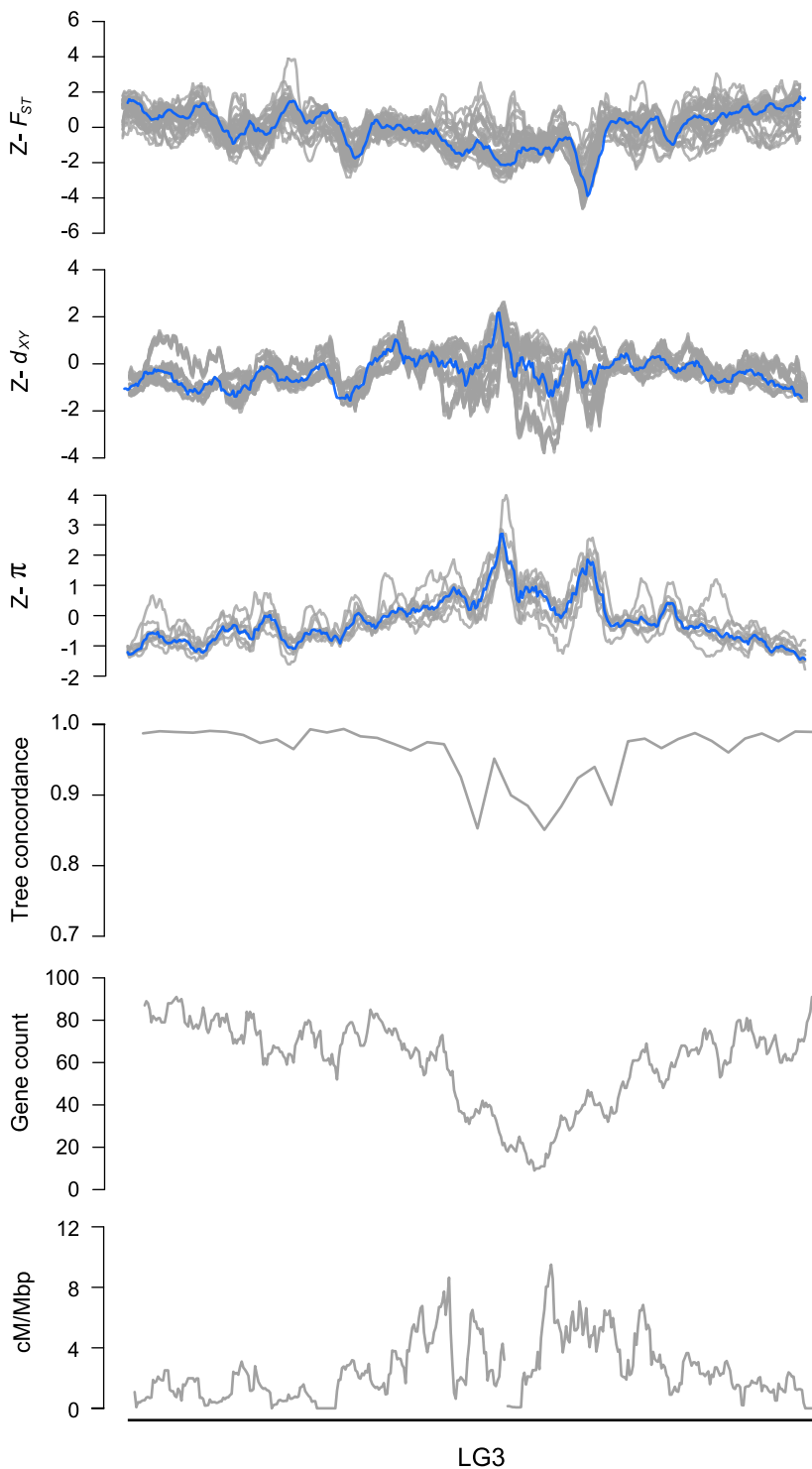

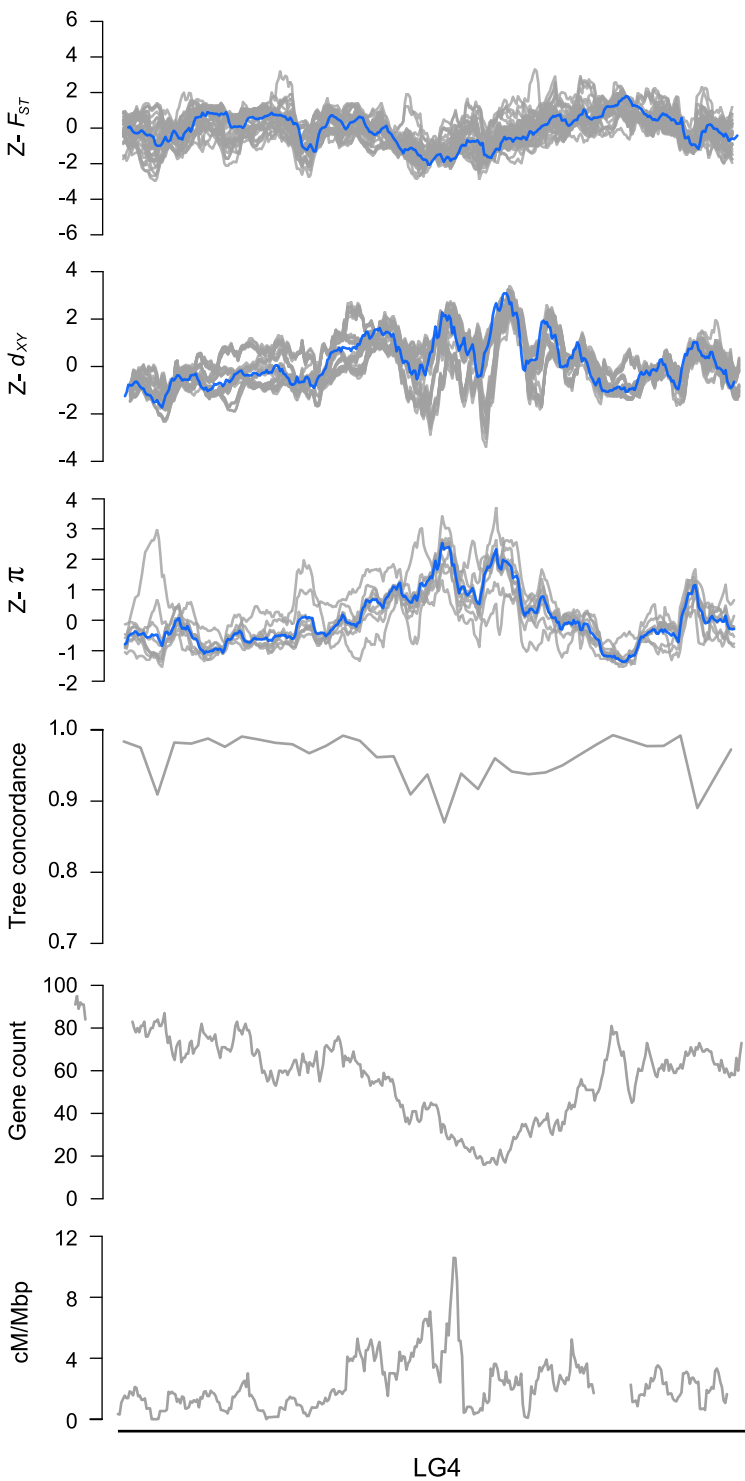

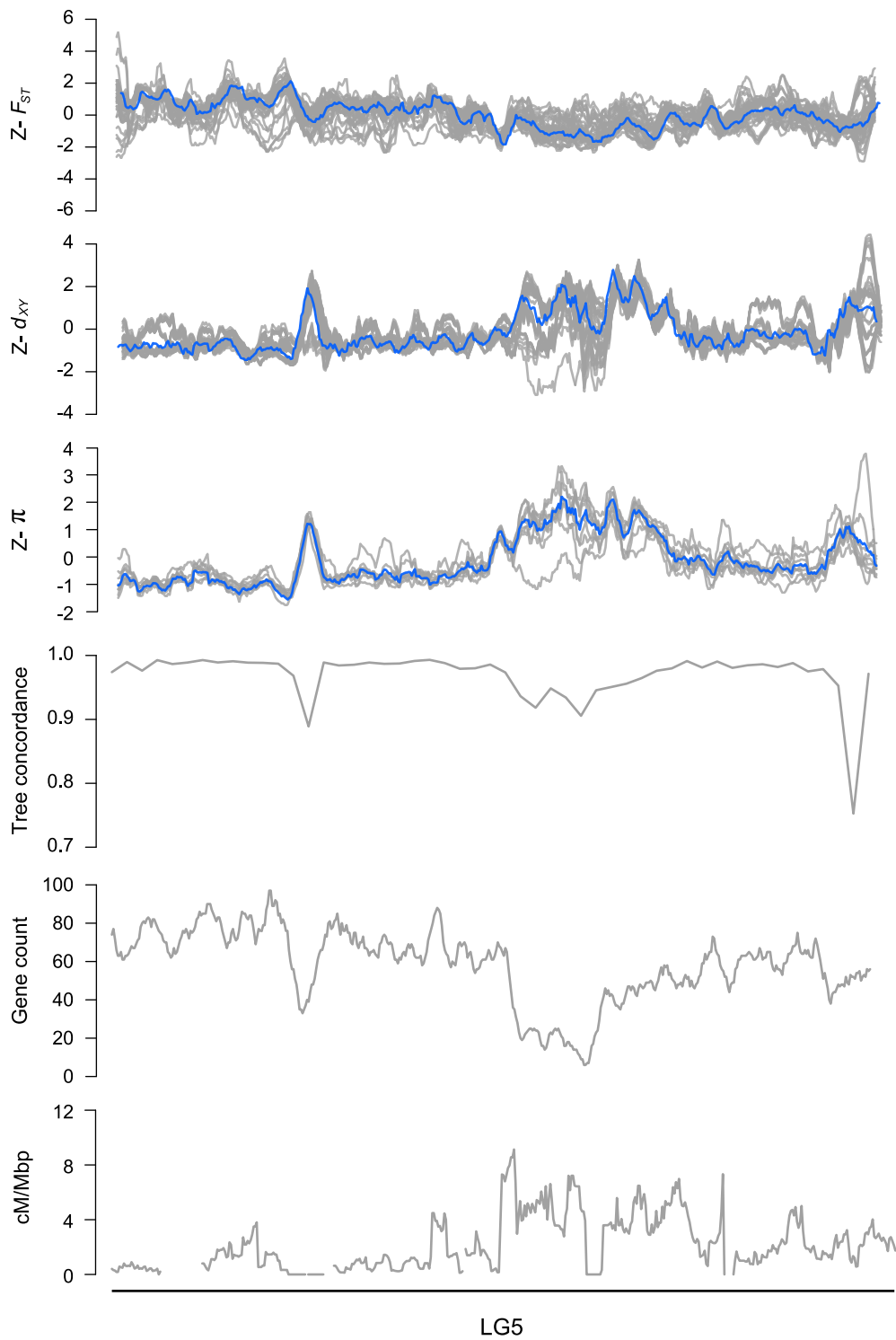

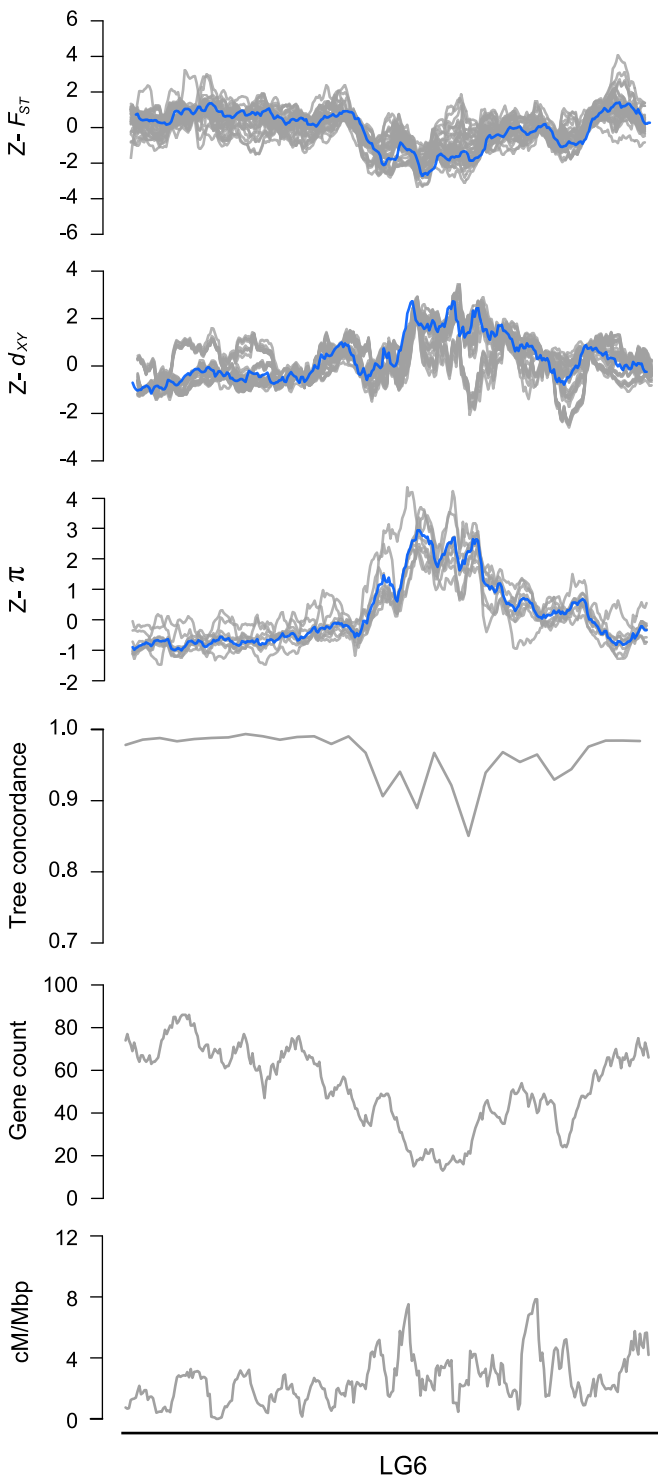

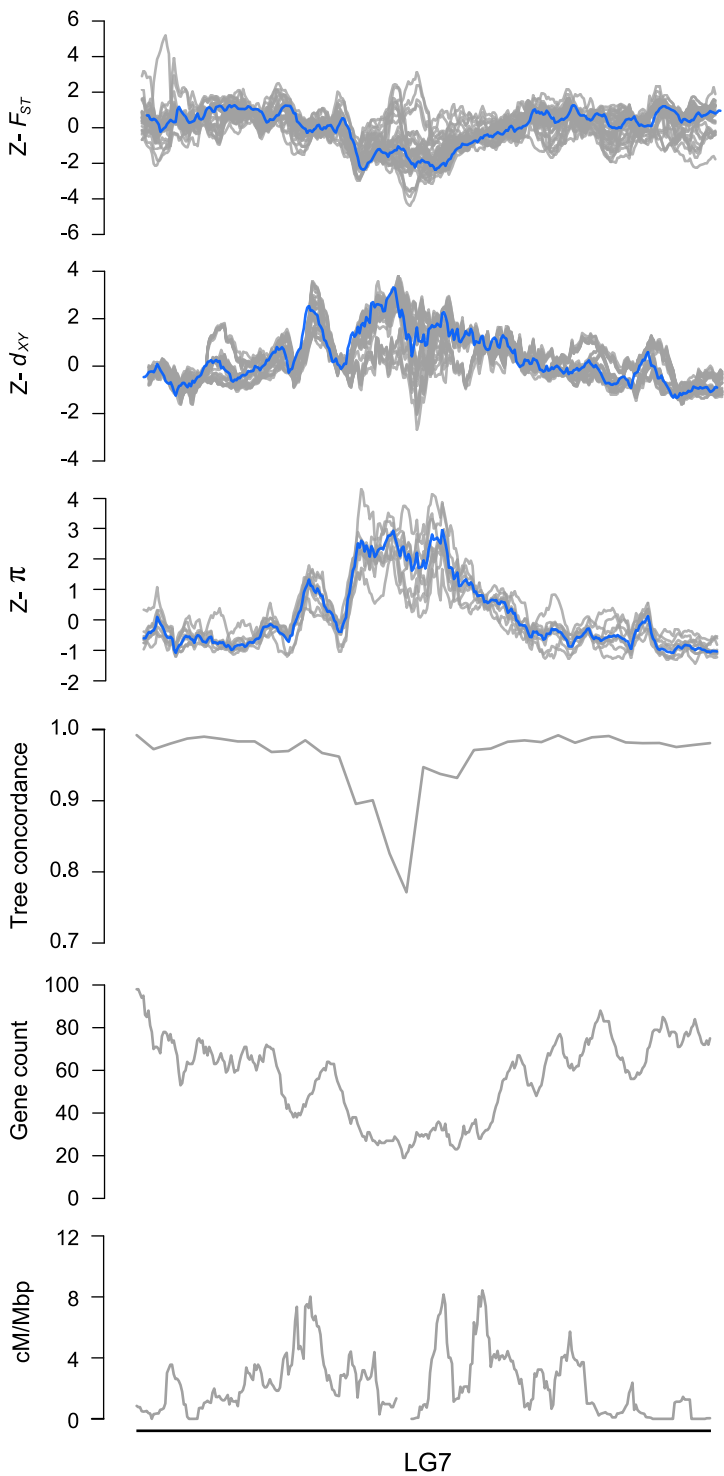

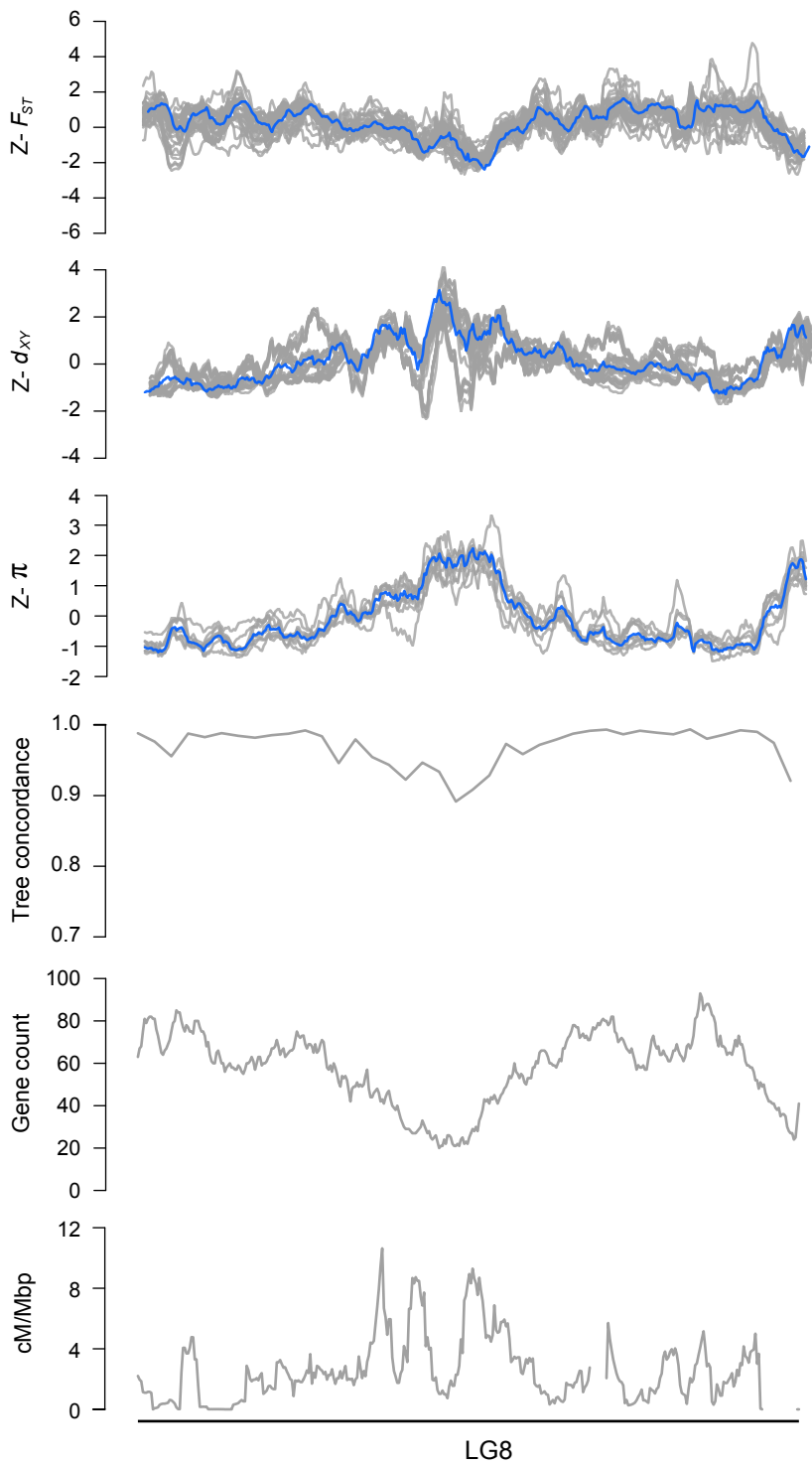

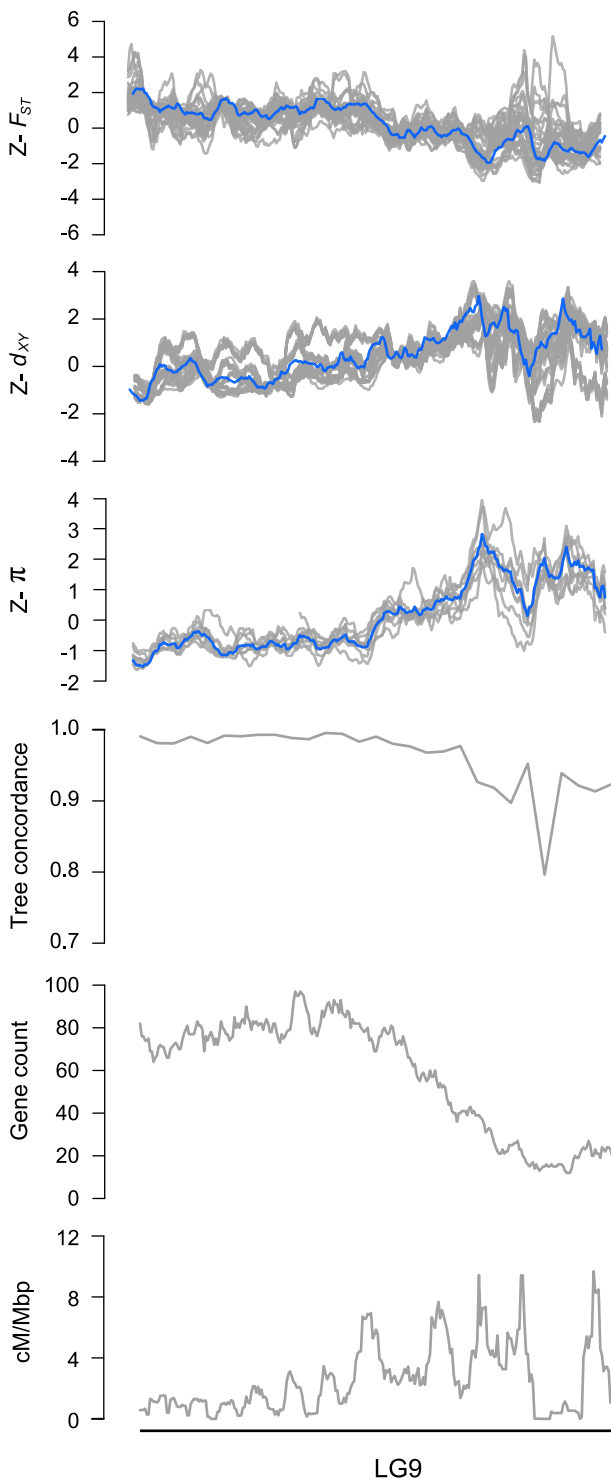

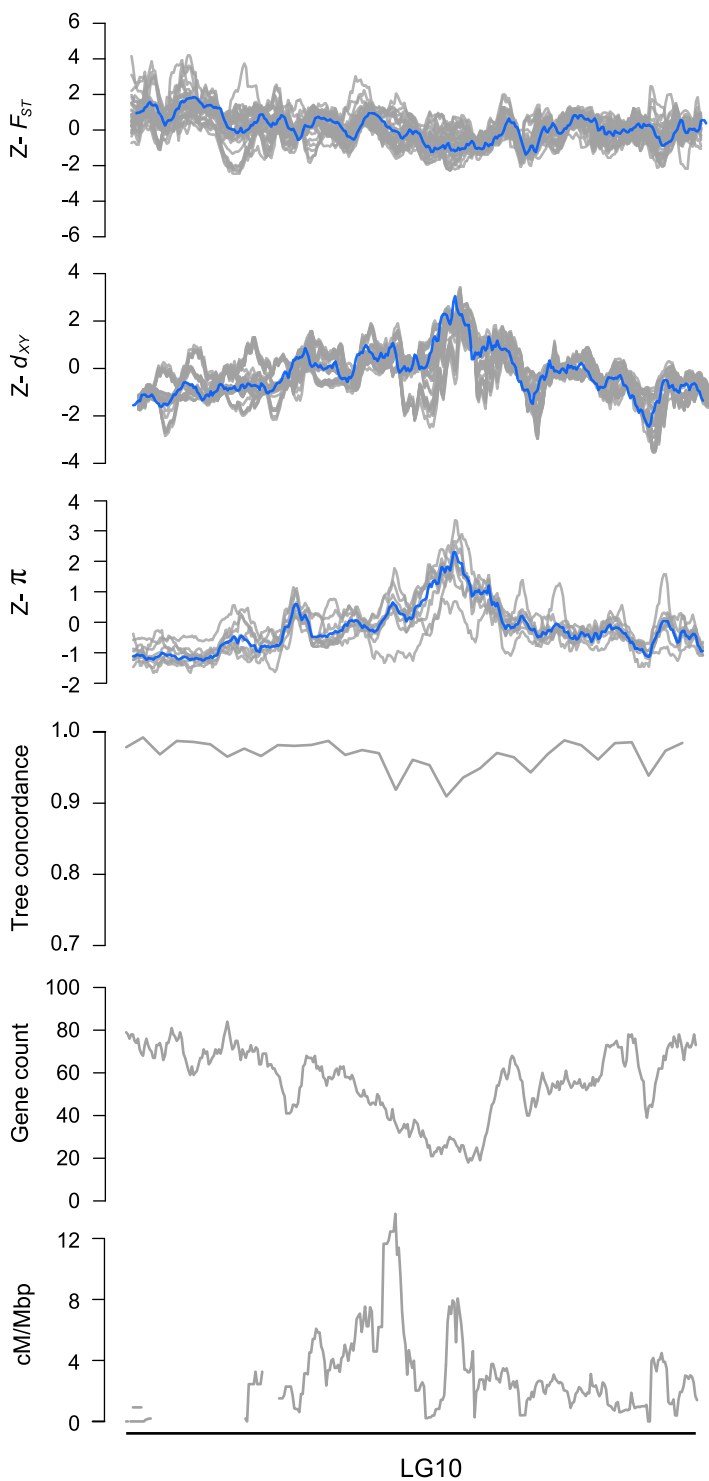

Supplement: S5 Fig — Z-transformed FST, dxy, and π in overlapping 500-kb windows (step size = 50 kbp). The gray lines are Z-transformed scores for each of the 36 pairwise comparisons (FST and dxy) or 9 taxa (π), and the blue line is the Z-transformed score for PC1. Estimates of tree concordance, gene count, and recombination rate (cM/Mbp) are also shown. LG, linkage group; PC1, first principal component. (PDF) [file pbio.3000391.s013.pdf]

$d_{xy} \text{ \& } \pi$  $F_{ST} \text{ \& } \pi$ 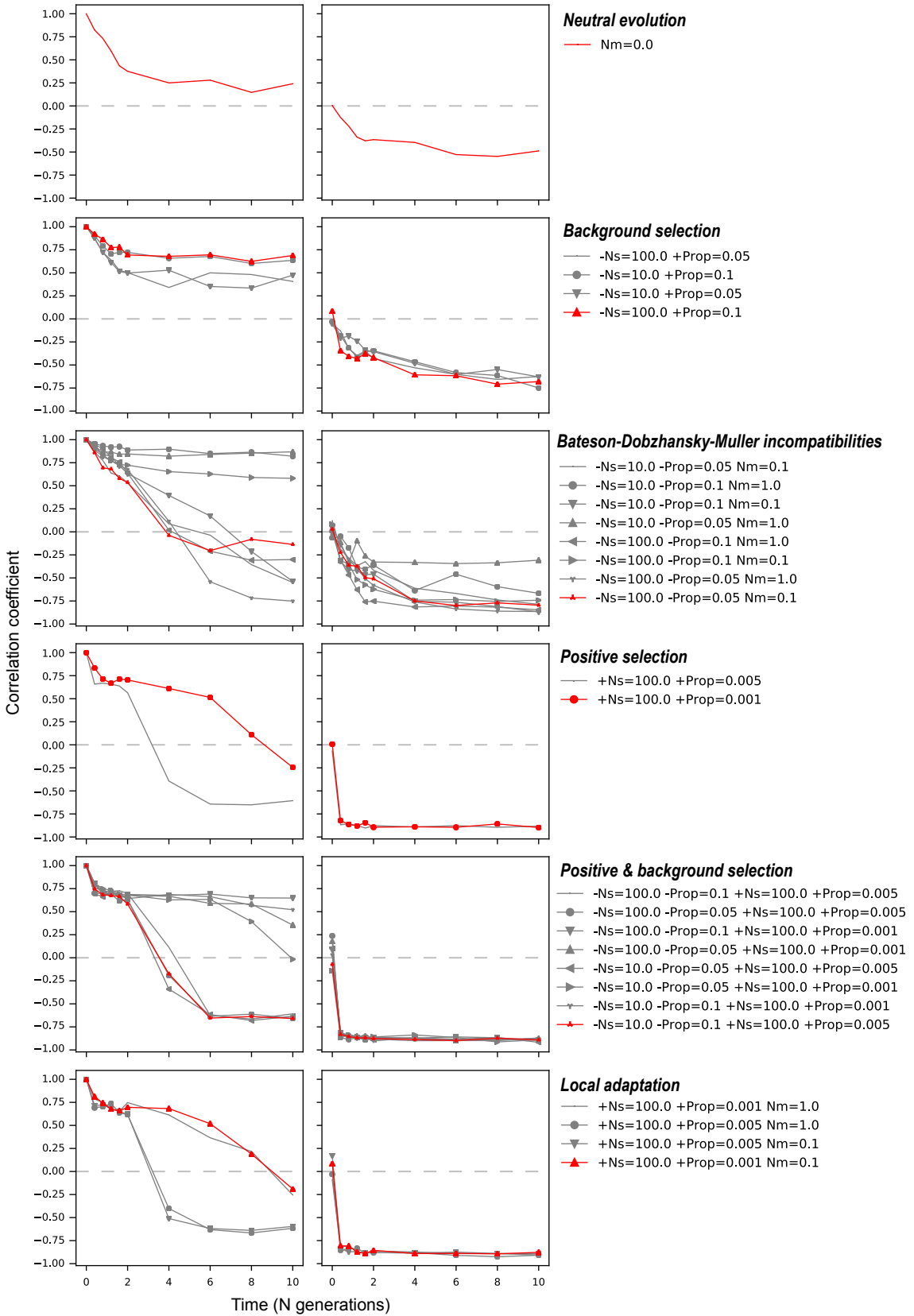

Supplement: S14 Fig — Each row of plots shows how the correlations between dxy and π (left plot) and FST and π (right plot) change over 10 time points (in N generations, where N = 10,000) in one of 6 scenarios: neutral divergence, BGS, BDMIs, positive selection, BGS and positive selection, and local adaptation. The red line in each plot shows the correlation for the simulation shown in Fig 7 of the main text. The gray lines show how correlations change for different combinations of parameter values in each scenario. The parameter Ns modulates the average selective coefficient (where s = Ns/N), whereas Prop is the proportion of new mutations that are not neutral. Nm is the average number of migrants per generation. Correlations represent the mean values estimated from 5 replicate simulations of each set of parameter values. BDMI, Bateson-Dobzhansky-Muller incompatibility; BGS, background selection. (PDF) [file pbio.3000391.s022.pdf]
