# Supplementary material for: Widespread selection and gene flow shape the genomic landscape during a radiation of monkeyflowers
Source: PLoS Biol. 2019 Jul 24;17(7):e3000391. doi: 10.1371/journal.pbio.3000391 (PMC6660095; doi:10.1371/journal.pbio.3000391)
Supplement: S13 Fig — Each row of plots shows patterns of within- and between-population variation (π, dxy, and FST) across a 21-Mb chromosome (500-kb windows) at 10 time points (in N generations, where N = 10,000) for one parameter combination of 6 scenarios: neutral divergence, BGS, BDMIs, positive selection, BGS and positive selection, and local adaptation. The gray boxes in the first column show the areas of the chromosome that are constrained by selection. Mean centered (above line) and raw values (below line) of π and dxy. The parameter Ns modulates the average selective coefficient (where s = Ns/N), whereas Prop is the proportion of new mutations that are not neutral. Nm is the average number of migrants per generation. BDMI, Bateson-Dobzhansky-Muller incompatibility; BGS, background selection. (PDF) [file pbio.3000391.s021.pdf]

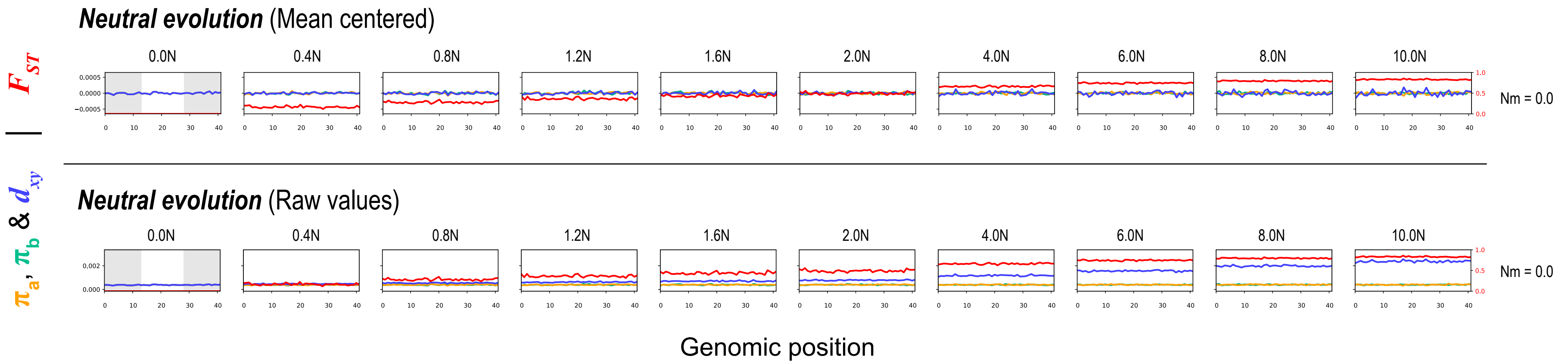

Neutral evolution (Mean centered)
Neutral evolution (Raw values)
0.0N
0.4N
0.8N
1.2N
1.6N
2.0N
4.0N
6.0N
8.0N
10.0N
Nm = 0.0
Genomic position

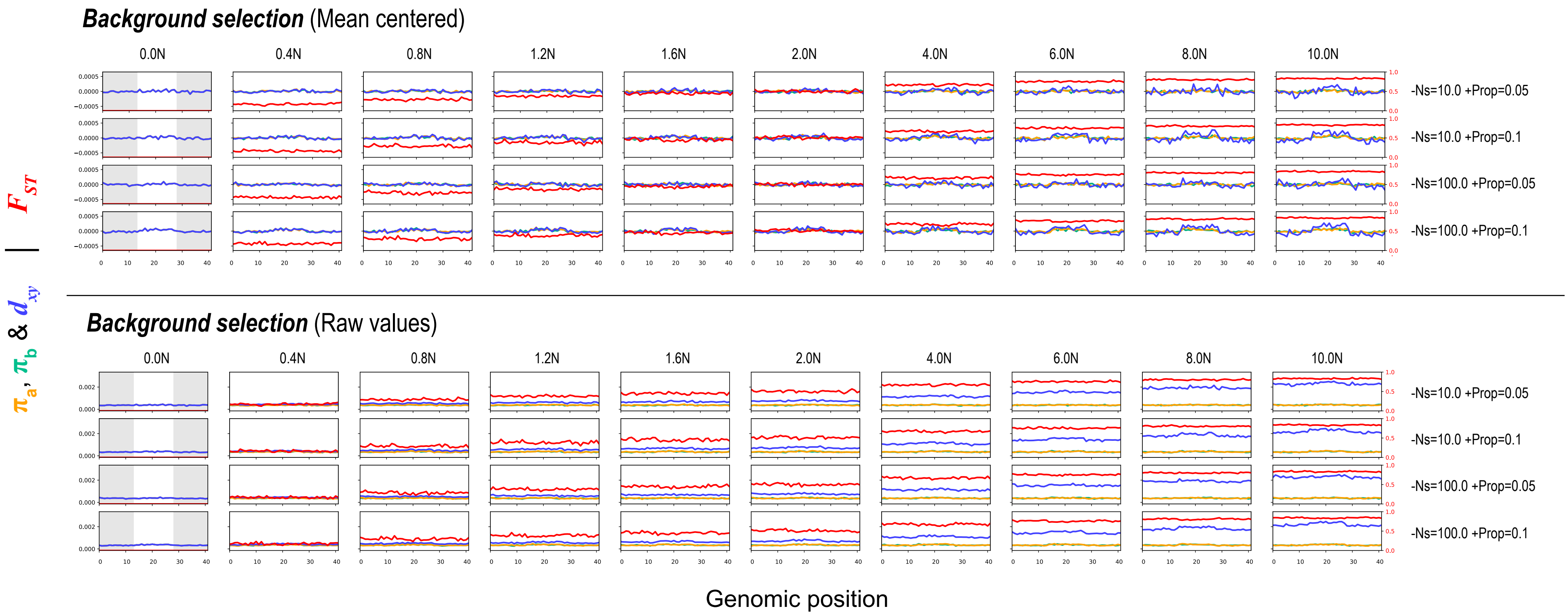

Background selection (Mean centered)
Background selection (Raw values)
0.0N
0.4N
0.8N
1.2N
1.6N
2.0N
4.0N
6.0N
8.0N
10.0N
-Ns=10.0 +Prop=0.05
-Ns=10.0 +Prop=0.1
-Ns=100.0 +Prop=0.05
-Ns=100.0 +Prop=0.1
F_{ST} | π_a, π_b & d_{xy}
Genomic position

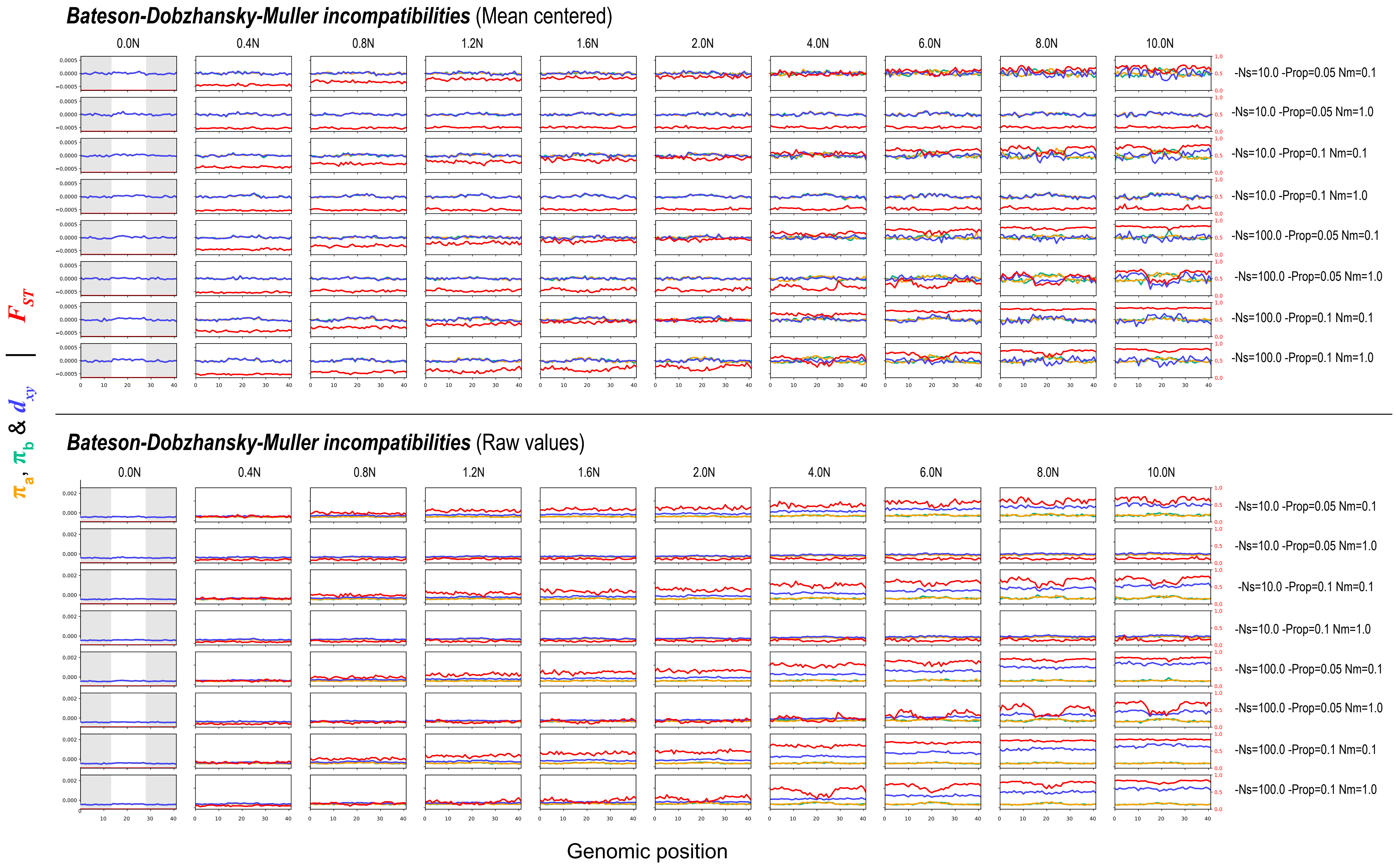

Bateson-Dobzhansky-Muller incompatibilities (Mean centered)
Bateson-Dobzhansky-Muller incompatibilities (Raw values)
0.0N
0.4N
0.8N
1.2N
1.6N
2.0N
4.0N
6.0N
8.0N
10.0N
-Ns=10.0 -Prop=0.05 Nm=0.1
-Ns=10.0 -Prop=0.05 Nm=1.0
-Ns=10.0 -Prop=0.1 Nm=0.1
-Ns=10.0 -Prop=0.1 Nm=1.0
-Ns=100.0 -Prop=0.05 Nm=0.1
-Ns=100.0 -Prop=0.05 Nm=1.0
-Ns=100.0 -Prop=0.1 Nm=0.1
-Ns=100.0 -Prop=0.1 Nm=1.0
π_a, π_b & d_{xy} | F_{ST}
Genomic position

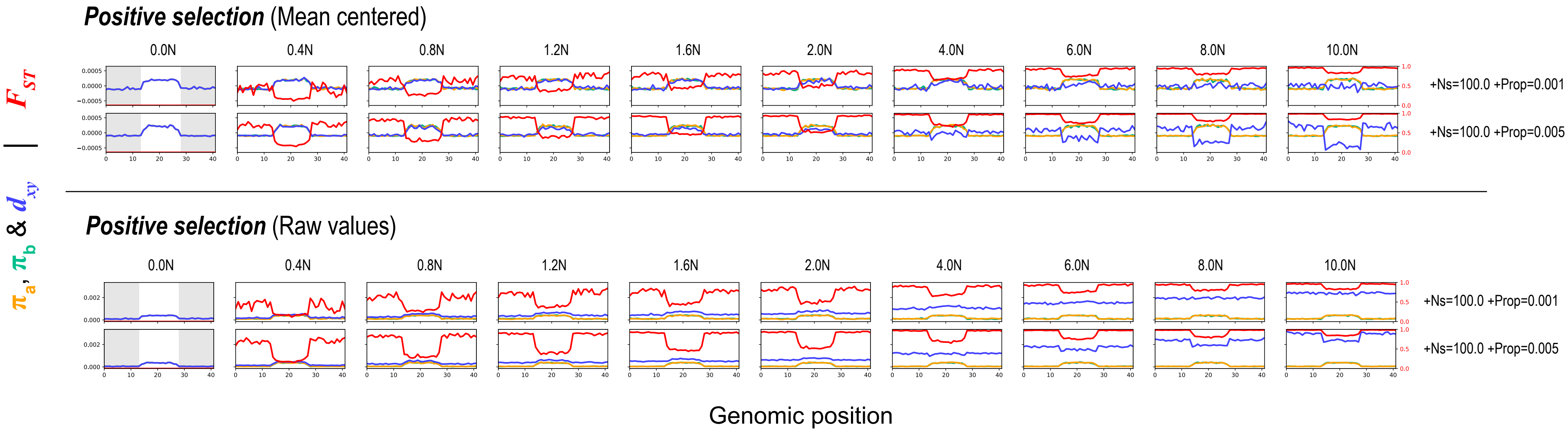

Positive selection (Mean centered)
Positive selection (Raw values)
0.0N
0.4N
0.8N
1.2N
1.6N
2.0N
4.0N
6.0N
8.0N
10.0N
+Ns=100.0 +Prop=0.001
+Ns=100.0 +Prop=0.005
F_{ST} | π_a, π_b & d_{xy}
Genomic position

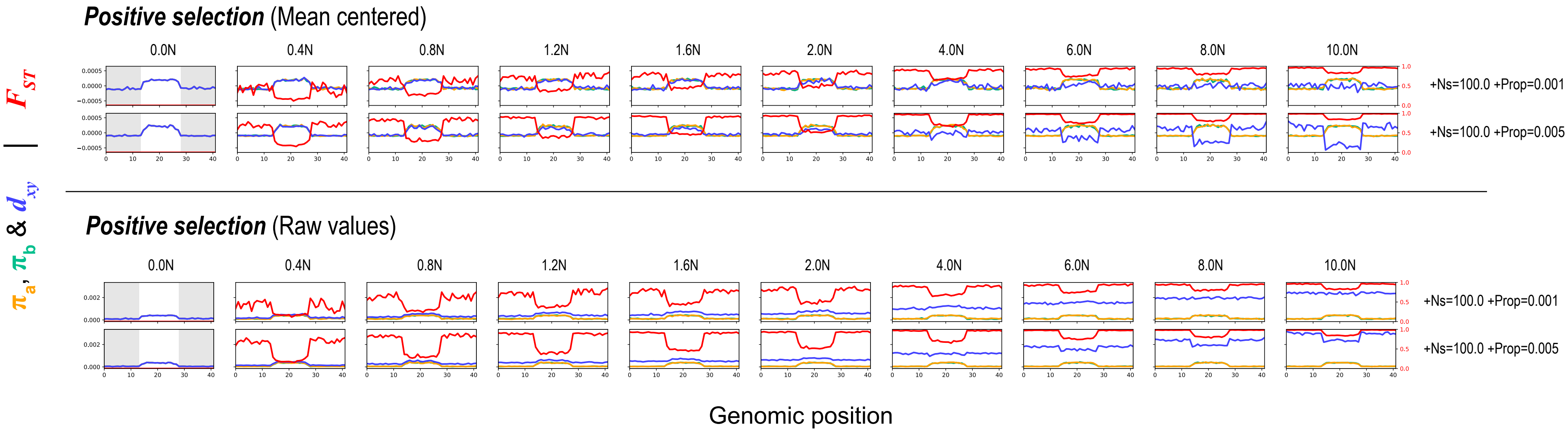

Positive selection (Mean centered)
Positive selection (Raw values)
0.0N
0.4N
0.8N
1.2N
1.6N
2.0N
4.0N
6.0N
8.0N
10.0N
+Ns=100.0 +Prop=0.001
+Ns=100.0 +Prop=0.005
F_{ST} | π_a, π_b & d_{xy}
Genomic position

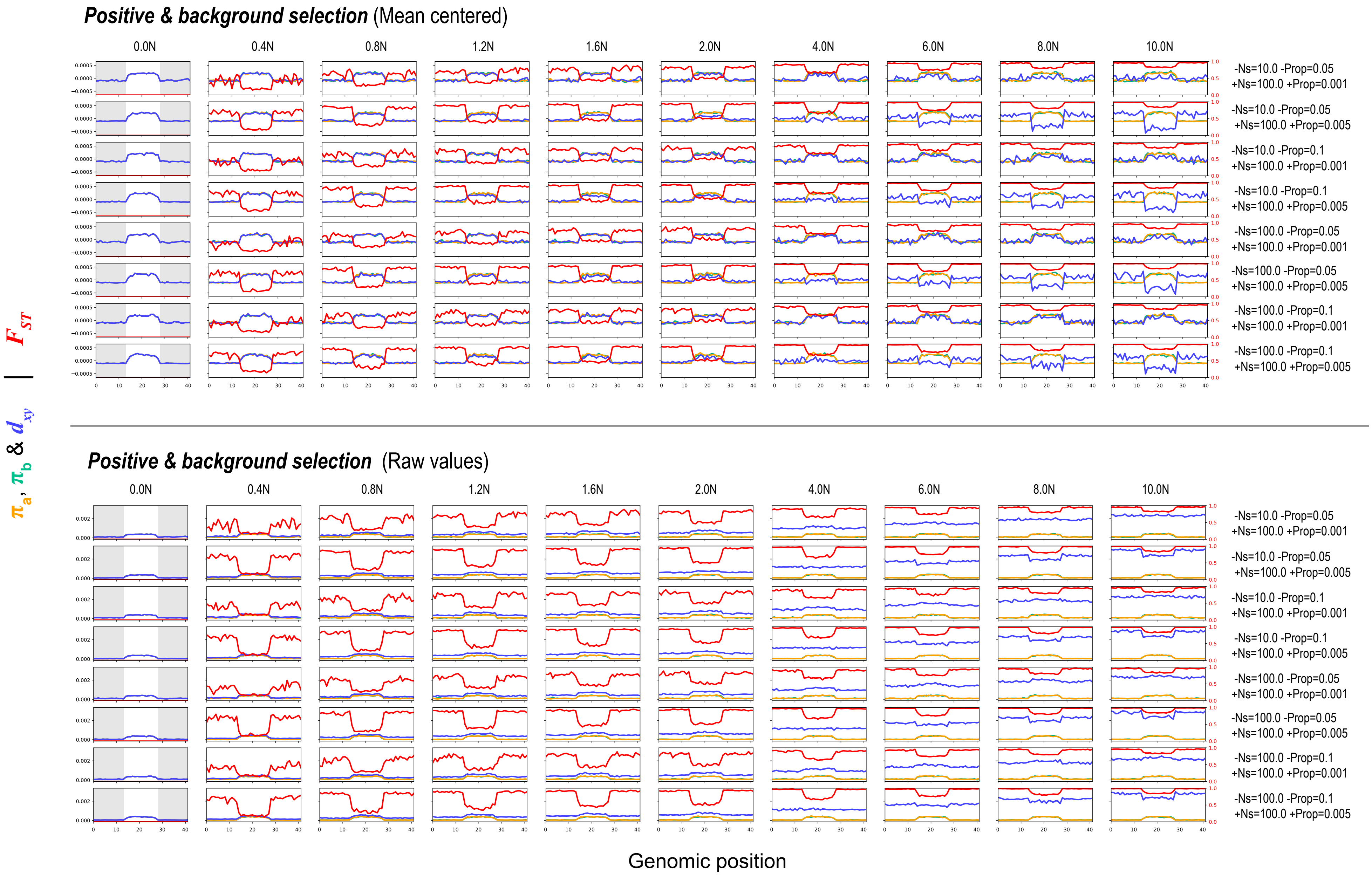
Positive & background selection (Mean centered)
Positive & background selection (Raw values)
0.0N
0.4N
0.8N
1.2N
1.6N
2.0N
4.0N
6.0N
8.0N
10.0N
-Ns=10.0 -Prop=0.05 +Ns=100.0 +Prop=0.001
-Ns=10.0 -Prop=0.05 +Ns=100.0 +Prop=0.005
-Ns=10.0 -Prop=0.1 +Ns=100.0 +Prop=0.001
-Ns=10.0 -Prop=0.1 +Ns=100.0 +Prop=0.005
-Ns=100.0 -Prop=0.05 +Ns=100.0 +Prop=0.001
-Ns=100.0 -Prop=0.05 +Ns=100.0 +Prop=0.005
-Ns=100.0 -Prop=0.1 +Ns=100.0 +Prop=0.001
-Ns=100.0 -Prop=0.1 +Ns=100.0 +Prop=0.005
F_{ST} | π_a, π_b & d_{xy}
Genomic position

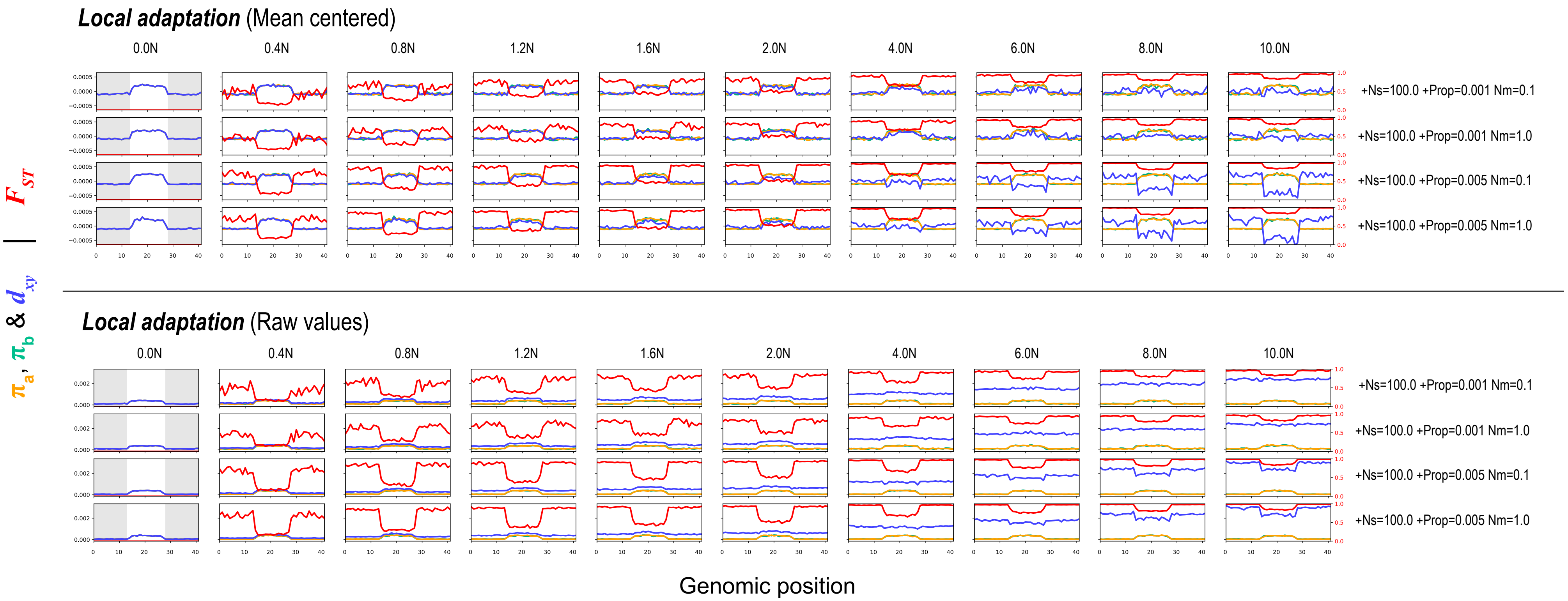

Local adaptation (Mean centered)
0.0N
0.4N
0.8N
1.2N
1.6N
2.0N
4.0N
6.0N
8.0N
10.0N
+Ns=100.0 +Prop=0.001 Nm=0.1
+Ns=100.0 +Prop=0.001 Nm=1.0
+Ns=100.0 +Prop=0.005 Nm=0.1
+Ns=100.0 +Prop=0.005 Nm=1.0
Local adaptation (Raw values)
0.0N
0.4N
0.8N
1.2N
1.6N
2.0N
4.0N
6.0N
8.0N
10.0N
+Ns=100.0 +Prop=0.001 Nm=0.1
+Ns=100.0 +Prop=0.001 Nm=1.0
+Ns=100.0 +Prop=0.005 Nm=0.1
+Ns=100.0 +Prop=0.005 Nm=1.0
F_{ST} | π_a, π_b & d_{xy}
Genomic position
